# Session: Evaluation of lung nodules: role of PET/CT

**DOI:** 10.1186/1470-7330-14-S1-O25

**Published:** 2014-10-09

**Authors:** Massimo Bellomi, Cristiano Rampinelli, Giulia Veronesi

**Affiliations:** 1 Department of Medical Imaging and Radiological Sciences, European Institute of Oncology. Via Ripamonti 435, 20141 Milano, Italy; 2 Department of Thoracic Surgery, European Institute of Oncology. Via Ripamonti 435, 20141 Milano, Italy

## 

Computed Tomographic (CT) screening for lung cancer remains controversial, although the National Lung Screening Trial (NLST) found that low dose CT (LDCT) reduced lung cancer mortality by about 20% [1], the high rate of indeterminate and false positive lung nodules was worrying.

The diagnostic work-up of CT nodules considers size (diameter or volume), characteristics (density, morphology, homogeneity) and volume doubling time (VDT), but several studies have investigated the ability of positron emission tomography (PET) to characterize nodules, although its role in diagnostic algorithms for screening-detected nodules has not been defined.

The first PET study with [^18^F]fluorodeoxyglucose to characterize indeterminate nodules outside screening was published in 2001 [2]. In a previous study on PET-CT at baseline screening, we found an overall sensitivity of 88% for diagnosing malignancy, while for solid nodules >10 mm, sensitivity was 100%, suggesting PET-CT as an alternative to invasive procedures in the screening setting [3]. When assessing the ability of PET-CT to diagnose indeterminate nodules detected during the subsequent years of the screening and analyzing 383 PET-CT examinations performed over 6 study years of screening, the sensitivity, specificity and accuracy of visually evaluated PET-CT, in distinguishing benign from malignant nodules, were 64%, 89% and 76% respectively. PET performance varied with nodule diameter (accuracy increased from 70% for nodules <10 mm to 82% for nodules ≥15 mm) and nodule type (accuracy ranged from 88% for solid nodules to 46% for non-solid nodules).

We do not use FNAB routinely in our screening protocol. Typically we proceed to surgery when nodule characteristics, including VDT, often backed up by PET-CT, indicate malignancy. The disadvantage is that a preoperative pathological diagnosis is not available and a wedge resection with frozen section examination is required before radical surgery.

Nodules on PET-CT are commonly evaluated by semi-quantitative SUVbw max, which is operator independent, as well as by visual assessment of uptake by the nuclear medicine physician, whose experience can markedly influence the result. In our study we evaluated both methods and found visual assessment afforded higher accuracy (76%) than any SUV threshold with a good compromise between sensitivity (64%) and specificity (89%); while increasing the threshold from 1.5 to 2.5 decreased sensitivity (67% to 51%) and increased specificity (80% to 91%).

To conclude, PET-CT has high NPV for solid nodules ≥15 mm, and high PPV for sub-solid nodules <10 mm, justifying its inclusion in the diagnostic work-up of indeterminate nodules identified on LDCT screening. It is more useful for nodules detected at baseline, while sensitivity is low for sub-solid nodules and nodules discovered after baseline: for these other diagnostic modalities, particularly VDT, are more useful.
